# Oral Mucositis Management in Children under Cancer Treatment: A Systematic Review

**DOI:** 10.3390/cancers16081548

**Published:** 2024-04-18

**Authors:** Ricardo Braguês, Maria Francisca Marvão, Patrícia Correia, Raquel M. Silva

**Affiliations:** 1 Universidade Católica Portuguesa, Faculty of Dental Medicine, 3504-505 Viseu, Portugal; s-rjandrade@ucp.pt (R.B.); francisca.marvao@hotmail.com (M.F.M.); pcorreia@ucp.pt (P.C.); 2 Universidade Católica Portuguesa, Center for Interdisciplinary Research in Health (CIIS), 3504-505 Viseu, Portugal

**Keywords:** pediatric cancer, oral mucositis, prevention, therapeutics, low-level laser therapy, palifermin, honey, zinc, calcium phosphate

## Abstract

**Simple Summary:**

Oral mucositis is a common and debilitating condition in pediatric patients undergoing antineoplastic therapy. Several interventions have been described for the management of oral mucositis; however, there is a lack of scientific evidence regarding their effectiveness and side effects in pediatric patients. This systematic review aimed to identify therapies for the prevention and treatment of oral mucositis in children, to assist clinicians in the decision-making process. Low-level laser therapy, palifermin, honey, and zinc demonstrated overall preventive effects on the development of oral mucositis, as well as improvement of symptoms.

**Abstract:**

Children undergoing antineoplastic treatment often present severe side effects due to the dosage and duration of treatments, with oral mucositis emerging as one of the most prevalent and painful inflammatory conditions. There is a growing body of evidence on therapeutic interventions such as cryotherapy, low-level laser therapy, and natural compounds for this condition. The aim of this systematic review was to identify and compare therapies for the management of cancer treatment-induced oral mucositis in pediatric patients. From 2655 articles obtained in initial searches, 39 articles were considered in this systematic review, after applying inclusion/exclusion criteria. Low-level laser therapy, palifermin, honey, and zinc demonstrated reductions in oral mucositis incidence, duration, severity, and pain reported by the patient. Although there are several therapies in place for the prevention and treatment of oral mucositis in children, evidence of their efficacy is still inconclusive to establish accurate clinical protocols.

## 1. Introduction

Improved supportive care and therapeutic regimens have contributed to increases in pediatric cancer survival rates and decreased the side effects associated with cancer therapy [1]. Antineoplastic therapy such as chemotherapy, radiotherapy, or a combination of both chemotherapy and radiotherapy are mostly used [2], but approaches like surgery, immunotherapy, and stem cell transplantation may also be necessary [3]. These treatments can cause moderate to severe side effects [4], including oral complications that are frequent in patients with childhood cancer. Maintaining good oral hygiene is indispensable, as well as assessing the patients’ oral health prior to and during antineoplastic treatment. Oral adverse effects include caries, periodontal disease [5], decreased salivary flow, dysgeusia (alterations in taste), dysphagia (difficulty in swallowing), oral cavity infections, spontaneous bleeding, osteoradionecrosis, trismus (difficulty in opening the mouth), neurotoxicity, and oral mucositis [6,7,8,9,10].

Oral mucositis is a debilitating condition defined as oral mucosa inflammation triggered by antineoplastic therapies [6]. Its incidence varies from 40% to 100%, depending on the type of malignancy, chemotherapy treatment, radiation dosage, age of the patient, oral health, and neutrophil count [5,11]. The chemotherapy drugs most often associated with the development of mucositis are doxorubicin, bleomycin, fluorouracil, and methotrexate, often used in therapeutic regimens for pediatric cancer patients [5,12], but the main clinical features are similar whether chemotherapy or radiotherapy takes place.

Oral mucositis is divided in five stages: initiation, signaling, amplification, ulceration, and healing, depending on its progression and the patient’s clinical presentation. Oral mucositis develops as reactive oxygen species that cause damage to the oral mucosa are generated, with activation of the transcription factor NF-kB, which increases production of pro-inflammatory cytokines. These further promote ulceration, enabling bacterial colonization and intensification of the pathogenic process [11]. In the early stages, oral mucositis appears as localized or generalized erythema, and the patient feels discomfort and a burning sensation when ingesting food. Over time, ulcerative and erosive lesions may occur, accompanied by extensive areas of necrosis and bleeding. As a result, patients feel pain and dysphagia, which may limit oral intake, and verbal communication is affected. These events lead to malnutrition and dehydration, and have a significant impact on the quality of life [11,12]. In addition to the associated morbidity, oral mucositis can interfere with the duration and intensity of antineoplastic treatment [12]. The most serious cases need to be hospitalized, to receive enteral or parenteral nutritional support, and often require reduction in the dosage or even suspension of therapy that can significantly alter cancer treatment and its prognosis. These patients are also at risk of sepsis development due to opportunistic infections, which can be fatal in immunosuppressed individuals [11,12]. 

Several interventions have been described for the management of oral mucositis; however, as none of the available therapeutic options can fully prevent or treat this condition, safe and effective treatment and prevention options are still necessary [13]. The clinical practice guidelines were recently reviewed for the use of oral cryotherapy; growth factors and cytokines such as keratinocyte growth factor (KGF) or palifermin; low-level laser therapy (LLLT) or photobiomodulation; anti-inflammatory agents, including oral rinses with benzydamine; and several antimicrobial, analgesic, and natural compounds [14,15]. One of these natural compounds is curcumin, which has obtained promising results in cell lines in vitro [16] and in animal models; however, there is limited information both in adults and children [17,18]. Despite these therapeutic interventions, there is lack of scientific evidence of their efficacy and side effects in pediatric patients [15]. Children are more susceptible to oral mucositis and not all treatments will be suitable for this population [11], as most studies have been performed on adults [14,15]. Therefore, the aim of this systematic review was to identify therapies for the prevention and treatment of oral mucositis specifically in pediatric patients, to provide guidance to clinicians in the decision-making process.

## 2. Materials and Methods

### 2.1. Protocol Registation 

This systematic review was registered in PROSPERO (International Prospective Register of Systematic Reviews), with the number CRD42022347208. The study was performed according to the PRISMA (Preferred Reporting Items for Systematic Reviews and Meta-Analyses) guidelines, which consist of a minimum set of items to improve reporting in systematic reviews, and followed the PICO (Patient, Intervention, Comparison and Outcome) strategy, which is used to frame clinical questions addressing the effect of an intervention or therapy. The research question defined was which treatments (I) are most effective (C) in the treatment and prevention (O) of cancer-induced oral mucositis in pediatric patients (P)?

### 2.2. Literature Search Strategy

Scientific literature and clinical trial searches were performed in PubMed/MEDLINE, NICE (National Institute for Health and Care Excellence), ICTRP (International Clinical Trials Registry Platform), Embase (MEDLINE excluded), Scopus, and Web of Science, up to March 2024. MeSH terms and Boolean operators “AND” and “OR” were applied in combination, referring to the population selected (pediatric patients under antineoplastic therapy that developed oral mucositis). The search was limited to human studies published in English or Portuguese, languages in which the authors were fluent, in the last 20 years to overcome potential bias stemming from outdated drugs or treatment protocols. The corresponding search terms for each platform are displayed in Supplementary Table S1. Search results were exported to the Rayyan tool for systematic literature reviews [19] to assist with duplicate removal and study selection, according to the inclusion and exclusion criteria defined and described below.

### 2.3. Exclusion and Inclusion Criteria

This systematic review was based on observational or experimental studies on the pediatric population (up to 18 years of age). Having completed or undergoing oncologic treatment was a requirement, as well as presenting outcomes for an oral mucositis therapeutic intervention. Secondary research articles such as systematic reviews and meta-analyses, narrative reviews, case reports, opinion articles, studies where children did not receive cancer treatment or did not develop oral mucositis, and studies that lacked an age range or control group were excluded. Studies in animal models or in vitro were also rejected (Supplementary Table S2). The titles and abstracts were screened by two independent investigators (R.B. and R.M.S.), followed by full-text assessment (R.B.). Articles were analyzed following the described exclusion and inclusion criteria. Any disagreement between investigators was discussed with a third investigator (P.C.) and solved by consensus. The Cohen’s kappa rating was 0.82 throughout the record screening phase, indicating almost perfect agreement between researchers (>99%).

### 2.4. Data Extraction and Quality Assessment

Data extraction and quality assessment were performed by one investigator (R.B.) and verified by two others (R.M.S. and P.C.). Data retrieved from selected articles included study identification and localization; study population data, such as sample size, patient age, and gender; and type of antineoplastic intervention (chemotherapy, radiotherapy, and/or hematopoietic stem cell transplantation), as well as the study methodology (case–control study, clinical trial, quasi-experimental, randomized controlled trial, retrospective study). The type of oral mucositis intervention, oral mucositis treatment duration, and outcomes (incidence, severity, duration, and pain reported by patients) were also collected. Quality assessment of the studies was performed with the Modified Newcastle–Ottawa Scale (NOS). The NOS is a star rating scale [20] that assigns a maximum of nine stars divided into three categories: selection of participants (0–4 stars), comparability (0–2 stars), and outcome/exposure (0–3 stars). Quality was determined numerically, from zero to nine points, according to the items indicated (Supplementary Table S3). High-quality articles were defined as those with a rating above five. The degree of bias was defined as high (≤5), moderate (6–7), or low (≥8). 

## 3. Results

### 3.1. Study Selection

**Figure 1:**
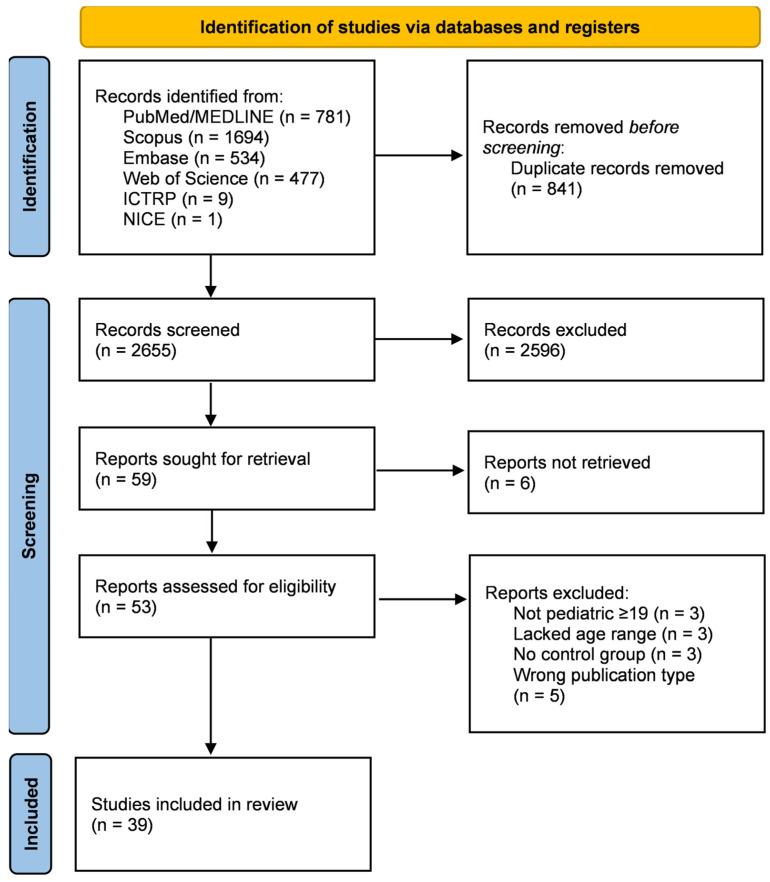
PRISMA flow diagram summarizing the study selection procedure, from the total number of records identified in the scientific literature database and clinical trial platform searches to the final studies included in this systematic review.

A total of 2655 unique records were identified through literature searches in the different scientific literature databases and clinical trial platforms, according to the strategy described in Supplementary Table S1. Of these, only 59 articles remained after reading the titles and abstracts and applying exclusion and inclusion criteria. Most excluded studies were performed on adult patients or included data from both children and adults (age ≥ 19 years). Others referred to oral mucositis prevalence with no therapeutic intervention for patient management or the outcome described. After full-text reading, 39 articles remained and were included in this systematic review [21,22,23,24,25,26,27,28,29,30,31,32,33,34,35,36,37,38,39,40,41,42,43,44,45,46,47,48,49,50,51,52,53,54,55,56,57,58,59]. The results are summarized in Figure 1. 

### 3.2. Characteristics of the Included Studies 

The antineoplastic therapies addressed in the selected studies were chemotherapy, radiotherapy, hematopoietic stem cell transplantation, or a combination of these. The malignant diseases and antineoplastic treatment details are provided in Supplementary Table S4. The sample size differed between studies; the lowest number of patients was 14 [29] and the highest was 148 [46]. Most studies were gender-balanced, although there was a slight excess of male patients in the samples. The study locations were distributed worldwide, with most of the studies performed in Brazil (eight articles), followed by Italy (six articles) and India (three articles) (Table 1).

**Figure 2:**
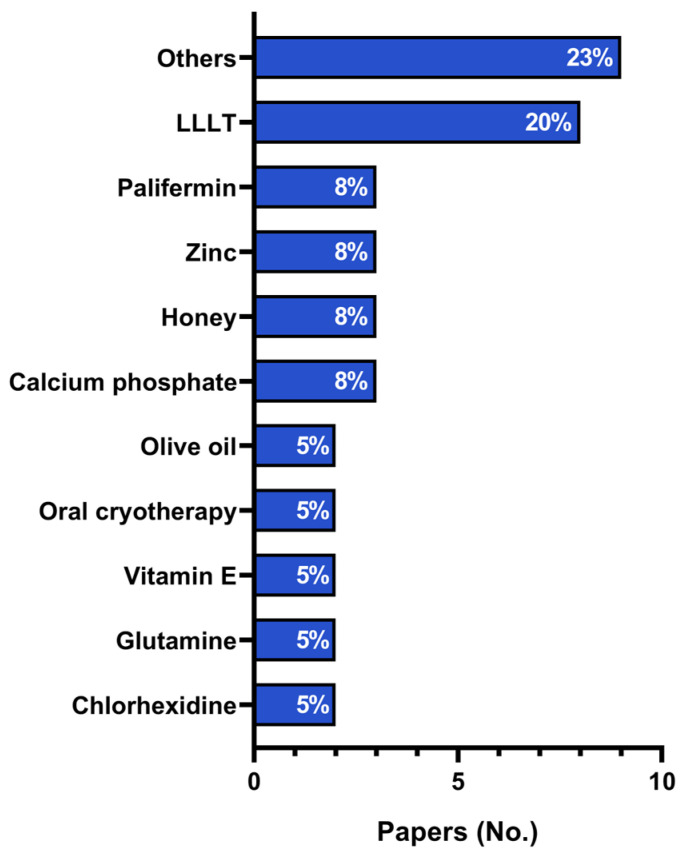
Most frequent therapies for oral mucositis management in the pediatric population undergoing antineoplastic treatment: LLLT (low-level laser therapy), chlorhexidine, calcium phosphate, honey, palifermin, oral cryotherapy, olive oil, vitamin E, glutamine, and zinc-containing compounds. “Others” include the following interventions: *Aloe vera*; andiroba orabase; bovine colostrum, chewing gum; high-power laser therapy; ketamine; Mucosamin^®^ oral spray; Mucosyte^®^ mouthwash; *Satureja hortensis* extract; transforming growth factor-beta 2; pycnogenol; and a mixture of honey, olive oil–propolis extract, and beeswax (one paper each).

The articles included in this systematic review described several therapies for oral mucositis management, including the most frequent LLLT [24,30,31,35,43,46,51,56] (20%), followed by palifermin [41,42,44], zinc-containing compounds [33,36,53], honey [21,25,40], calcium phosphate [37,45,49] (8%), olive oil [23,25], oral cryotherapy [38,52], vitamin E [39,57], glutamine [28,59], and chlorhexidine [29,47] (5%) (Figure 2). Interventions that were considered in only one study included *Aloe vera* [22]; andiroba orabase [55]; bovine colostrum [50]; chewing gum [34]; high-power laser therapy [58]; ketamine [48]; Mucosamin^®^ oral spray [54]; Mucosyte^®^ mouthwash [26]; *Satureja hortensis* gel [27]; transforming growth factor-beta 2 [32]; pycnogenol [39]; and a mixture of honey, olive oil–propolis extract, and beeswax [21]. Oral mucositis treatment duration varied from 3 to 29 days (Table 2). Additional data, such as oral mucositis grade and treatment dose, are provided in Supplementary Table S4.

### 3.3. Therapeutic Efficacy of Each Intervention

**Figure 3:**
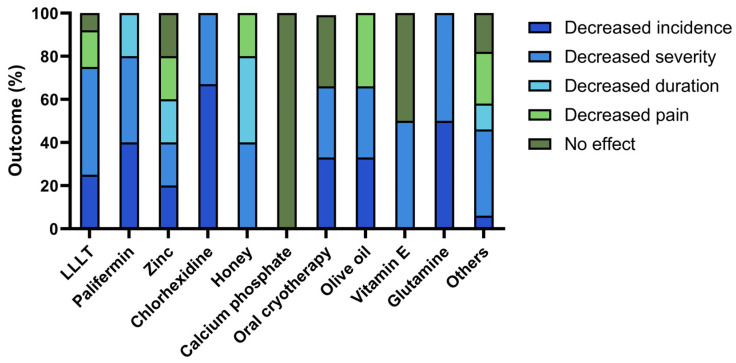
Therapeutic efficacy of each intervention, assessed by a decrease in the incidence, severity, duration, and pain associated with oral mucositis in the pediatric population undergoing antineoplastic treatment. LLLT, low-level laser therapy.

Most therapies showed some degree of improvement of oral mucositis, leading to a reduction in its incidence, duration, severity, or pain reported by the patient. However, it is important to note that not all articles reported the same level of therapeutic efficacy (Figure 3). Calcium phosphate had no effect on oral mucositis symptoms, whereas chlorhexidine and glutamine were reported to decrease both the incidence and severity of oral mucositis. Similarly, palifermin reduced the incidence, severity, and duration of oral mucositis, while honey decreased the severity, duration, and pain associated with it. Olive oil decreased incidence, severity, and pain. Zinc-based compounds and other treatments had all outcomes, including some reports with no effects. LLLT, oral cryotherapy, and vitamin E also had reports without effects on oral mucositis outcomes.

The most effective treatment for oral mucositis in the pediatric population was chlorhexidine, reported as decreasing its incidence in the studies included in this systematic review. Considering oral mucositis severity, chlorhexidine, honey, palifermin, olive oil, vitamin E, and glutamine showed similar results in the studies describing decreases in oral mucositis severity with these interventions. Honey was the most effective treatment in decreasing the duration of oral mucositis. To reduce pain, olive oil had the highest efficacy, while honey, LLLT, and zinc-based compounds were equally effective (Figure 3).

### 3.4. Quality Assessement

The Modified Newcastle–Ottawa Scale, which assesses the quality of study methodology, established high bias in 9 articles [28,29,31,33,38,41,44,47,56] and low bias in 14 articles [21,22,23,25,32,35,42,43,45,48,50,51,53,59]. The remaining articles had moderate bias [24,26,27,30,34,36,37,39,40,46,49,52,54,55,57,58]. Studies with a high risk of bias had small sample sizes overall [29,33,44,56], or small number of patients in either the intervention or control groups [28,47]. Study design [29,31,38,56] and low patient acceptance [28,38] were also considered as bias factors (Supplementary Table S3).

## 4. Discussion

Pediatric patients undergoing antineoplastic treatment are particularly susceptible to oral mucositis. Oral mucositis decreases the patient’s quality of life and can potentially impact cancer therapy, increasing the risk of fatality [38]. This study presents the existing evidence on the management of oral mucositis secondary to cancer treatment in children. These findings are clinically relevant to support the existing guidelines. 

### 4.1. Low-Level Laser Therapy 

LLLT, or photobiomodulation, is the application of a low-power red and near-infrared light without raising the tissue temperature [60]. The therapeutic effects of LLLT include the promotion of collagen synthesis and fibroblast production and reductions in inflammation and pain [60,61]. LLLT was reported to benefit patients with oral mucositis, either alone or in combination with cryotherapy [61] or photochemotherapy [43]; however, there is no standard protocol available for the pediatric population [60]. In this systematic review, most studies addressed LLLT in the management of oral mucositis in children, reporting a decrease in incidence, severity, or pain associated with this condition [24,31,35,43,46,51,56]. Only the oldest study from this group did not find an effect of LLLT in oral mucositis [30], but differences in light wavelength, light exposure, and application techniques made the results challenging to compare [60,62]. Notably, five of these studies were carried out in Brazil [30,31,43,46,56], two in Italy [24,35], and the one remaining in Egypt [51]. Most of the studies were performed on patients under chemotherapy regimens, and recent guidelines recommend LLLT for pediatric patients undergoing hematopoietic stem cell transplant or radiotherapy [63]. The effectiveness of LLLT in the pediatric population is not fully understood, but it may be due to its ability to reduce oxidative stress and pro-inflammatory cytokines that are associated with oral mucositis development [60,61]. Extraoral light application might increase adherence to LLLT in children refusing intraoral protocols [56,60].

### 4.2. Palifermin

Palifermin is a recombinant human keratinocyte growth factor (KGF) that binds to the KGF receptor expressed in epithelial cells of the epidermis, pancreas, liver, lung, and urothelium [41]. In epithelial cells expressing the KGF receptor, palifermin stimulates cell proliferation, differentiation, and protection against apoptotic mechanisms [41,42]. Palifermin can decrease the incidence, severity, and duration of oral mucositis in patients with acute leukemia, and its administration is considered safe and without significant complications [41,42,44]. The epithelial DNA damage and oxidative stress prevented by palifermin might underlie its effectiveness in children who have developed oral mucositis, but a recent report has highlighted possible adverse side effects and does not recommend its use [63]. However, Morris et al. found no clinically important alterations in the liver, blood, metabolic, or enzymatic profiles in patients during the palifermin treatment period. This study determined a safe and tolerable dose of palifermin in pediatric patients in different age groups and showed a good safety profile [44]. No novel reports have been published since, which was also acknowledged in a recent review that addressed the management of oral mucositis patients and included both pediatric and adult populations [64]. The latest systematic review and meta-analysis on the management of oral mucositis in children supports the use of palifermin [65], but further studies are needed to assess the use of palifermin as a safe and efficient intervention for oral mucositis treatment and prevention in children.

### 4.3. Zinc

Zinc is a trace metal with antioxidant properties that acts on cell division, tissue repair, immunity, and protein and DNA synthesis [36,53]. There are several zinc supplements, such as zinc gluconate and polaprezinc, but their differences in oral mucositis management remain to be defined [33,36,53]. Zinc gluconate was reported to reduce severity, duration, and pain in children with oral mucositis [36]; however, a new study found no effect in the pediatric population [53]. These studies implemented different therapeutic doses of zinc gluconate, 50 mg and 1 mg/kg/day (maximum 30 mg), respectively, and further studies are needed to determine an effective dosage for oral mucositis management in children. Polaprezinc or zinc L-carnosine suspension in sodium alginate were effective in decreasing the incidence of oral mucositis [33]. Although polaprezinc is safe [66], several children refused the intervention with this compound because of an unpleasant flavor and texture [33]. Polaprezinc’s taste needs to be improved, as its effectiveness in reducing oral mucositis in children is promising due to its wound-healing and antioxidant properties [33,67]. Further studies are needed to compare the differences between the therapeutic effects of zinc gluconate and polaprezinc. 

### 4.4. Chlorhexidine

Chlorhexidine mouthwash is an antimicrobial agent used in the prophylactic management of oral mucositis [68,69]. Chlorhexidine was the most effective therapeutic intervention to reduce oral mucositis incidence in children under cancer treatment [29,47]. Although these are the oldest studies in our systematic review, published in 2003 and 2006, chlorhexidine mouthwashes are already part of oral care protocols for the management of pediatric patients with oral mucositis, namely, in severe cases when brushing cannot be supported [70]. The preventive removal of potentially pathogenic oral bacteria is probably responsible for the effectiveness of chlorhexidine mouthwash in children, as the oral microflora has a role in the pathogenesis of oral mucositis development [68,69].

### 4.5. Honey

Honey is a natural substance with anti-inflammatory and antimicrobial properties that can aid in the process of healing wounds and ulcers, either with topical or systemic use [25]. Topical application of natural honey, produced from flower nectar, has been reported to decrease the severity and duration of oral mucositis in the pediatric population [21,40]. In comparison, manuka honey, collected from manuka tree pollen, has been found to be able to decrease both the severity and pain associated with oral mucositis in children [25]. In this study, olive oil showed similar results to honey, although its taste was unpleasant for children [25]. Natural honey is less expensive than manuka honey, although both are safe and can be used to manage pediatric oral mucositis, as reported also in a recent review [71]. Honey’s pleasant taste increases children’s adherence to the therapy, and its effectiveness in oral mucositis management is due to the decrease in pro-inflammatory cytokines and elimination of pathogenic oral bacterial colonization [25,40].

### 4.6. Calcium Phosphate

Calcium phosphate or Caphosol^®^ mouth rinses are suggested to improve the healing of oral mucosal lesions by reducing inflammation and promoting epithelial proliferation [37]. No effect was observed for calcium phosphate rinses in the outcomes regarding oral mucositis in the three studies considered in this systematic review [37,45,49]. This evidence is supported by clinical practice guidelines suggested to manage oral mucositis in cancer patients, although these are not limited to studies in the pediatric population [67]. The ineffectiveness of calcium phosphate as a therapy for oral mucositis in the pediatric population is not entirely comprehended [49], and further studies are needed to address the influence of this compound on oral mucositis development.

### 4.7. Oral Cryotherapy

Oral cryotherapy is a method of cooling the mouth during chemotherapy sessions. It promotes local vasoconstriction, leading to reduced absorption of chemotherapeutic agents by the oral mucosa, resulting in less tissue toxicity [38,52]. There is evidence that oral cryotherapy can reduce the incidence and severity of oral mucositis in adults, but studies in the pediatric population are limited [38]. In this systematic review, oral cryotherapy showed a reduction in the incidence and severity of oral mucositis in children when administered with propantheline [52], but no effect when administered alone [38,52]. The ineffectiveness of oral cryotherapy administration in children may be related to low compliance, as several children refused the intervention due to discomfort or nausea [38,52]. Replacement of plain ice cubes with flavored ice cubes may increase children’s compliance to oral cryotherapy [52], but further studies are required.

### 4.8. Olive Oil

Olive oil is a natural substance produced from the fruit of the olive tree (*Olea europaea*), with anti-inflammatory and antibacterial properties that can promote wound healing when administered topically or systemically [21,22,25]. In this systematic review, topical application of olive oil was the most effective therapeutic intervention to decrease pain associated with oral mucositis in children [25]. Olive oil was also reported to decrease the incidence and severity of oral mucositis in the pediatric population [23,25], showing similar results to honey, although children reported an unpleasant taste [25]. The effectiveness of olive oil in oral mucositis management in children may be related to the inhibition of pro-inflammatory cytokines in the acute stage of oral mucositis development, although this is not fully understood [23]. Further studies should aim to improve the taste of olive oil for application in oral mucositis therapeutic interventions.

### 4.9. Vitamin E

Vitamin E has antioxidant properties capable of protecting cellular membranes from oxidative stress, and has been studied against side effects of cancer treatment [57]. One study included in this systematic review found that vitamin E had no effect in the outcomes of oral mucositis [57], while Khurana et al. reported decreased oral mucositis severity in the pediatric population [39]. It is possible that the ineffectiveness of vitamin E is related to patient characteristics, as the oldest study had a small percentage of intervention cycles with pediatric patients who developed severe oral mucositis [57]. Further studies should address the effectiveness of vitamin E on the outcomes of oral mucositis in children.

### 4.10. Glutamine

Glutamine improves the immune system and promotes the production of hexosamine, a substance that coats the mucosa and acts as a barrier [59]. Prevention of oral mucositis with glutamine has been used in children with acute lymphoblastic leukemia after high-dose methotrexate chemotherapy. After a glutamine dose of 400 mg/kg/day (oral or intravenous), a significant reduction in the incidence of oral mucositis was reported in both studies, with few side effects [28,59]. The intravenous administration of glutamine may increase the compliance of children, as oral medication intake might be impaired due to pain associated with oral mucositis [28].

### 4.11. Other Therapies

Administration of TGF-β2 [32], chewing gum [34], or ketamine mouthwash [48] had no effect on the outcomes of oral mucositis in children. Additional interventions, such as the use of 70% *Aloe vera* solution [22]; Mucosyte^®^ mouthwash [26]; pycnogenol [39]; bovine colostrum [50]; Mucosamin^®^ oral spray [54]; 3% andiroba orabase [55]; high-power laser therapy [58]; 1% *Satureja hortensis* gel [27]; and a mixture of honey, olive oil–propolis extract, and beeswax [21] reduced one or several outcomes (incidence, severity, duration, or pain) of oral mucositis in children, although there was only one article for each in our dataset.

### 4.12. Limitations and Future Directions

Some studies included in this systematic review had limitations due to small sample sizes [29,33,43,57,58], sample heterogeneity, study design biases [31,33,39,59], or low adherence to treatment [25,33,38,52]. The risk-of-bias grading (high, moderate, and low) using the Modified Newcastle–Ottawa Scale revealed a high [28,29,31,33,38,41,44,47,56] to moderate risk in certain papers [24,26,27,30,34,36,37,39,40,46,49,52,54,55,57,58]. Equally, the diversity of cancer types and variations in clinical protocols, as well as oral mucositis treatment duration, could constitute confounding factors. Additionally, the measurement of diverse outcomes, such as incidence, severity, duration, and pain associated with oral mucositis, may have also limited the comparability between the efficacy levels of the therapies studied in this systematic review. Future research should overcome these limitations by increasing sample sizes, improving oral mucositis data collection, and addressing outcomes measures.

## 5. Conclusions

Cryotherapy requires further studies in the pediatric population, since it lacks scientific evidence in this age group. For palifermin, there are reports both in favor and against its use in children; thus, further studies are needed to support it as a therapeutic option. LLLT alone or in combination with photochemotherapy favors healing and reduces infections in the oral cavity, improving the patients’ quality of life. The use of glutamine, olive oil, and honey is also safe, and chlorhexidine is effective in controlling oral mucositis in the pediatric population. 

Oral mucositis emerges as a challenge to pediatric oncologists and pediatric dentists. An adequate and regular assessment of the oral cavity by the pediatric dentist, as part of the health care team, is important in order to diagnose this condition and determine the best intervention. Robust evidence in the literature is essential to support clinical decision making on the best treatment options to prevent and treat oral mucositis in children. 

## Figures and Tables

**Table 1:** Characteristics of the studies included in this systematic review. Study type, sample size, age range and mean, antineoplastic intervention, and location of the study are indicated.

Author and Publication Year	Study Type	Sample Size	Age Range and Mean (Years)	Antineoplastic Intervention	Location	Ref.
Abdulrhman M et al., 2012	RCT	90 (57 M, 33 F)	2–18 (6.9 ± 3.8)	CT	Egypt	[21]
Alkhouli M et al., 2021	RCT	22 (14 M, 8 F)	3–6 (4.6)	CT	Syria	[22]
Alkhouli M et al., 2019	RCT	22 (12 M, 10 F)	4–6 (5.4)	CT	Syria	[23]
Amadori F et al., 2016	RCT	123 (56 M, 67 F)	3–18 (9.8 ± 3.3)	CT or HSCT	Italy	[24]
Badr LK et al., 2023	RCT	42 (23 M, 19 F)	5–17 (10.9 ± 4.1)	CT	Lebanon	[25]
Bardellini E et al., 2016	RCT	56 (22 M, 34 F)	5–18 (7.0 ± 1.8)	CT	Italy	[26]
Bostanabad MA et al., 2018	RCT	60 (40 M, 20 F)	3–14 (7.6 ± 3.4)	CT	Iran	[27]
Chang YH et al., 2017	Retrospective	96 (49 M, 37 F)	0–18 (8.8 ± 5.2)	CT	Taiwan	[28]
Costa EM et al., 2003	Clinical trial	14 (n.a.)	2–10 (7.0)	CT	Brazil	[29]
Cruz LB et al., 2007	RCT	60 (39 M, 21 F)	3–18 (8.7 ± 4.3)	CT or HSCT	Brazil	[30]
de Castro JFL et al., 2013	Clinical trial	40 (27 M, 13 F)	1–18 (6.8)	CT	Brazil	[31]
de Koning BA et al., 2007	RCT	25 (17 M, 8 F)	0–18 (8.0)	CT	Netherlands	[32]
Funato M et al., 2018	Retrospective	16 (9 M, 7 F)	1–18 (7.2)	HSCT	Japan	[33]
Gandemer V et al., 2007	RCT	145 (93 M, 52 F)	5–18 (11.6)	CT	France	[34]
Gobbo M et al., 2018	RCT	101 (54 M, 47 F)	3–18 (11.9)	CT	Italy	[35]
Gutiérrez-Vargas R et al., 2020	Quasi-experimental	49 (29 M, 20 F)	8–16 (11.1 ± 2.7)	CT	Mexico	[36]
Immonen E et al., 2020	RCT	45 (25 M, 20 F)	2–18 (6.5)	CT	Finland	[37]
Kamsvåg T et al., 2020	RCT	49 (26 M, 23 F)	4–17 (11.3 ± 3.8)	HSCT	Sweden	[38]
Khurana H et al., 2013	RCT	72 (57 M, 15 F)	6–15 (9.3 ± 2.6)	CT	India	[39]
Kobya HB et al., 2016	Quasi-experimental	76 (38 M, 38 F)	6–18 (10.9 ± 4.1)	CT	Turkey	[40]
Lauritano D et al., 2014	Case-control study	40 (21 M, 19 F)	7–16 (11.0)	RT	Italy	[41]
Lucchese A et al., 2016	RCT	54 (26 M, 28 F)	7–16 (11.0)	HSCT	Italy	[42]
Medeiros-Filho JB et al., 2017	RCT	15 (14 M, 1 F)	3–16 (9.5)	CT and CT + RT	Brazil	[43]
Morris J et al., 2016	Clinical trial	27 (15 M, 12 F)	1–16 (8.5)	CT + RT	United States	[44]
Mubaraki S et al., 2020	RCT	45 (20 M, 25 F)	7–10 (7.7 ± 3.1)	HSCT	Saudi Arabia	[45]
Nunes LFM et al., 2020	Retrospective	148 (82 M, 66 F)	1–17 (9.2 ± 4.6)	HSCT	Brazil	[46]
Pinto LP et al., 2006	Clinical trial	33 (n.a.)	2–15 (8.5)	CT	Brazil	[47]
Prakash S et al., 2020	RCT	44 (35 M, 9 F)	8–18 (11.5 ± 2.9)	CT	India	[48]
Raphael MF et al., 2014	RCT	29 (19 M, 10 F)	4–18 (11.3 ± 3.9)	CT or HSCT	Netherlands	[49]
Rathe M et al., 2020	RCT	62 (32 M, 30 F)	1–18 (5)	CT	Denmark	[50]
Reyad D et al., 2022	RCT	44 (21 M, 23 F)	2–14 (7.4 ± 2.5)	CT	Egypt	[51]
Sato A et al., 2006	Retrospective	24 (19 M, 5 F)	2–16 (7.0)	CT	Japan	[52]
Shah D et al., 2023	RCT	90 (n.a.)	3–18 (6.0)	CT	India	[53]
Shahrabi M et al., 2022	RCT	60 (n.a.)	4–18 (11.9 ± 5.5)	HSCT	Iran	[54]
Soares ADS et al., 2021	RCT	60 (n.a.)	6–12 (9.0)	CT	Brazil	[55]
Soto M et al., 2015	Clinical trial	24 (17 M, 7 F)	2–16 (7.9)	HSCT	Brazil	[56]
Sung L et al., 2007	RCT	16 (10 M, 6 F)	6–18 (12.7)	CT	Canada	[57]
Vitale M et al., 2017	RCT	16 (n.a.)	3–18 (10.5)	CT or HSCT	Italy	[58]
Widjaja NA et al., 2020	RCT	48 (31 M, 17 F)	1–18 (6.3)	CT	Indonesia	[59]

RCT, randomized controlled trial; M, male; F, female; CT, chemotherapy; HSCT, hematopoietic stem cell transplantation; RT, radiotherapy; n.a., not available.

**Table 2:** Oral mucositis treatments, duration, and outcomes in the studies considered in this systematic review.

Author and Publication Year	Oral Mucositis Treatment Type	Oral Mucositis Treatment Duration (Days)	Oral Mucositis Outcome	Ref.
Abdulrhman M et al., 2012	Honey	10	Decreased duration	[21]
	Mixture of honey, olive oil–propolis extract, and beeswax	10	Decreased duration	
Alkhouli M et al., 2021	70% *Aloe vera*	(n.a.)	Decreased severity	[22]
Alkhouli M et al., 2019	Olive oil	(n.a.)	Decreased incidence and decreased severity	[23]
Amadori F et al., 2016	LLLT	4	Decreased pain	[24]
Badr LK et al., 2023	Manuka honey	7	Decreased severity and decreased pain	[25]
	Olive oil	7	Decreased pain	
Bardellini E et al., 2016	Mucosyte^®^ mouthwash	8	Decreased severity and decreased pain	[26]
Bostanabad MA et al., 2018	*Satureja hortensis* extract mucoadhesive gel of 1%	5	Decreased pain	[27]
Chang YH et al., 2017	Parenteral glutamine	3	Decreased incidence and decreased severity	[28]
Costa EM et al., 2003	0.12% Chlorhexidine	10	Decreased incidence and decreased severity	[29]
Cruz LB et al., 2007	LLLT	5	No effect	[30]
de Castro JFL et al., 2013	LLLT	5	Decreased incidence and decreased severity	[31]
de Koning BA et al., 2007	TGF-β2	(n.a.)	No effect	[32]
Funato M et al., 2018	Polaprezinc sodium alginate suspension	(n.a.)	Decreased incidence	[33]
Gandemer V et al., 2007	Chewing gum	3	No effect	[34]
Gobbo M et al., 2018	LLLT	4	Decreased severity and decreased pain	[35]
Gutiérrez-Vargas R et al., 2020	Zinc gluconate	(n.a.)	Decreased severity, decreased duration and decreased pain	[36]
Immonen E et al., 2020	Calcium phosphate rinse (Caphosol^®^)	7	No effect	[37]
Kamsvåg T et al., 2020	Oral cryotherapy	13	No effect	[38]
Khurana H et al., 2013	Vitamin E	7	Decreased severity	[39]
	Pycnogenol	7	Decreased severity	
Kobya HB et al., 2016	Honey	21	Decreased severity and decreased duration	[40]
Lauritano D et al., 2014	Palifermin	21	Decreased severity	[41]
Lucchese A et al., 2016	Palifermin	3–6	Decreased incidence, decreased severity and decreased duration	[42]
Medeiros-Filho JB et al., 2017	LLLT and PCT	8	Decreased severity	[43]
Morris J et al., 2016	Palifermin	6	Decreased incidence	[44]
Mubaraki S et al., 2020	Calcium phosphate rinse	(n.a.)	No effect	[45]
Nunes LFM et al., 2020	LLLT	(n.a.)	Decreased incidence and decreased severity	[46]
Pinto LP et al., 2006	0.12% Chlorhexidine	10	Decreased incidence	[47]
Prakash S et al., 2020	Ketamine mouthwash	(n.a.)	No effect	[48]
Raphael MF et al., 2014	Calcium phosphate rinse (Caphosol^®^)	(n.a.)	No effect	[49]
Rathe M et al., 2020	Bovine colostrum	29	Decreased severity	[50]
Reyad F et al., 2022	LLLT	4	Decreased severity	[51]
Sato A et al., 2006	Oral cryotherapy and propantheline	(n.a.)	Decreased incidence and decreased severity	[52]
Shah D et al., 2023	Zinc gluconate syrup	14	No effect	[53]
Shahrabi M et al., 2022	Mucosamin^®^ oral spray	14	Decreased incidence, decreased severity and decreased duration	[54]
Soares ADS et al., 2021	3% Andiroba (*Carapa guianensis*) orabase	11	Decreased severity and decreased pain	[55]
Soto M et al., 2015	LLLT	22	Decreased incidence and decreased severity	[56]
Sung L et al., 2007	Vitamin E	14	No effect	[57]
Vitale M et al., 2017	HPLT	4	Decreased severity and decreased pain	[58]
Widjaja NA et al., 2020	Oral glutamine	14	Decreased incidence and decreased severity	[59]

LLLT, low-level laser therapy; TGF-β2, transforming growth factor-beta 2; PCT, photochemotherapy; HPLT, high-power laser therapy; n.a., not available.

## Data Availability

Data sharing is not applicable.

## Supplementary Materials

The following supporting information can be downloaded at: https://www.mdpi.com/article/10.3390/cancers16081548/s1, Supplementary Table S1: Literature search with the corresponding search terms in each database; Supplementary Table S2: Inclusion and exclusion criteria; Supplementary Table S3: Quality assessment analysis using the Modified Newcastle–Ottawa Scale; Supplementary Table S4: Additional characteristics of the included studies.
