# Supplementary material for: Oral Mucositis Management in Children under Cancer Treatment: A Systematic Review
Source: Cancers (Basel). 2024 Apr 18;16(8):1548. doi: 10.3390/cancers16081548 (PMC11048343; doi:10.3390/cancers16081548)
Supplement: Supplementary file 1 [file cancers-16-01548-s001.zip › cancers-2930271-supplementary.pdf]

SUPPLEMENTARY MATERIAL

Supplementary Table S1. Literature search with the corresponding search terms in each database.

PubMed/MEDLINE ®	(((((("oral mucositis"[All Fields] OR "oral cavity"[All Fields] OR "oral mucosa"[All Fields] OR "oral complications"[All Fields] OR "oral mucosa injuries"[All Fields] OR "stomatitis"[MeSH Terms]) AND ("child"[MeSH Terms] OR "child"[All Fields] OR "children"[All Fields] OR "child s"[All Fields] OR "children s"[All Fields] OR "childrens"[All Fields] OR "childs"[All Fields] OR "pre schooler*"[All Fields] OR "pre schooler*"[All Fields] OR ("childhood"[All Fields] OR "childhoods"[All Fields]) OR "pre teen*"[All Fields] OR "preteen*"[All Fields] OR "teen*"[All Fields] OR "teenager*"[All Fields] OR ("adolescent"[MeSH Terms] OR "adolescent"[All Fields] OR "youth"[All Fields] OR "youths"[All Fields] OR "youth s"[All Fields]) OR "paediatric patient*"[All Fields] OR "adolescen*"[All Fields] OR "paediatric population"[All Fields] OR "child"[MeSH Terms] OR "child, preschool"[MeSH Terms] OR ("child, preschool"[MeSH Terms] OR ("child"[All Fields] AND "preschool"[All Fields]) OR "preschool child"[All Fields] OR "preschooler"[All Fields] OR "preschoolers"[All Fields] OR "preschool"[All Fields] OR "preschooler s"[All Fields] OR "preschools"[All Fields]) OR "adolescent"[MeSH Terms]) AND ((((((("chemotherapy s"[All Fields] OR "drug therapy"[MeSH Terms] OR ("drug"[All Fields] AND "therapy"[All Fields]) OR "drug therapy"[All Fields] OR "chemotherapies"[All Fields] OR "drug therapy"[MeSH Subheading] OR "chemotherapy"[All Fields]) AND "cancer treatment"[All Fields]) AND ()) AND "cancer therap*"[All Fields]) AND ()) AND "drug therap*"[All Fields]) OR "radiation therap*"[All Fields] OR "oncological therap*"[All Fields] OR ("radiate"[All Fields] OR "radiated"[All Fields] OR "radiates"[All Fields] OR "radiating"[All Fields] OR "radiation"[MeSH Terms] OR "radiation"[All Fields] OR "electromagnetic radiation"[MeSH Terms] OR ("electromagnetic"[All Fields] AND "radiation"[All Fields]) OR "electromagnetic radiation"[All Fields] OR "radiations"[All Fields] OR "radiation s"[All Fields] OR "radiator"[All Fields] OR "radiators"[All Fields]) OR "radiation injur*"[All Fields] OR "therap*"[All Fields] OR "radiotherapy"[MeSH Terms] OR "therapy"[MeSH Subheading] OR "antineoplastic agents"[MeSH Terms] OR ("radiation"[MeSH Terms] OR "electromagnetic radiation"[MeSH Terms]) OR "therapeutics"[MeSH Terms]) AND ("cancer s"[All Fields] OR "cancerated"[All Fields] OR "canceration"[All Fields] OR "cancerization"[All Fields] OR "cancerized"[All Fields] OR "cancerous"[All Fields] OR "neoplasms"[MeSH Terms] OR "neoplasms"[All Fields] OR "cancer"[All Fields] OR "cancers"[All Fields] OR ("cysts"[MeSH Terms] OR "cysts"[All Fields] OR "cyst"[All Fields] OR "neurofibroma"[MeSH Terms] OR "neurofibroma"[All Fields] OR "neurofibromas"[All Fields] OR "tumor s"[All Fields] OR "tumoral"[All Fields] OR "tumorous"[All Fields] OR "tumour"[All Fields] OR "neoplasms"[MeSH Terms] OR "neoplasms"[All Fields] OR "tumor"[All Fields] OR "tumour s"[All Fields] OR "tumoural"[All Fields] OR "tumourous"[All Fields] OR "tumours"[All Fields] OR "tumors"[All Fields]) OR "neoplasms"[MeSH Terms])) NOT ("review"[Publication Type] OR "review literature as topic"[MeSH Terms] OR "review"[All Fields])) NOT ("systematic review"[Publication Type] OR "systematic reviews as topic"[MeSH Terms] OR "systematic review"[All Fields])) NOT ("meta analysis"[Publication Type] OR "meta analysis as

	topic"[MeSH Terms] OR "meta analysis"[All Fields])) NOT ("case reports"[Publication Type] OR "case reports"[All Fields])) AND ((humans[Filter]) AND (english[Filter] OR portuguese[Filter]) AND (2003:2024[pdat]))
NICE	Oral mucositis treatment AND Cancer AND Children
ICTRP	Oral mucositis treatment AND Cancer AND Children
Embase (MEDLINE excluded)	("oral mucositis of oral cavity" or "oral mucosa" or "oral complications" or "oral mucosa injuries" or stomatitis).mp. [mptitle, abstract, heading word, drug trade name, original title, device manufacturer, drug manufacturer, device trade name, keyword heading word, floating subheading word, candidate term word] and (children or "pre-schooler" or "preschooler or childhood or "pre teen" or "preteen" or teen" or teenager" or youth or "paediatric patient or adolescen" or "paediatric population" or child or "child preschool" or preschool or adolescent).mp.(mp-title,abstract, heading word, drug trade name, original title, device manufacturer, drug manufacturer, device trade name, keyword heading word, floating subheading word, candidate term word] and (Chemotherapy or "cancer treatment" or "cancer therap" or "drug therap" or "radiation therap" or "oncological therap" or radiation or "radiation injur" or "therap" Radiotherapy" or "antineoplastic agents" or Therapeutics).mp.(mp-title, abstract, heading word, drug trade name, original title, device manufacturer, drug manufacturer, device trade name, keyword heading word, floating subheading word, candidate term word] and (cancer or tumour or neoplasms).mp. (mp-title, abstract, heading word, drug trade name, original title, device manufacturer, drug manufactures, device trade name, keyword heading word, floating subheading word, candidate term word] limit to (human and (english or portuguese) and yr=”2003 - 2024”) and limit to “remove medline records”
Scopus	(TITLE-ABS-KEY (({oral mucositis} OR {oral cavity} OR {oral mucosa} OR {oral complications} OR {oral mucosa injuries} OR {stomatitis})) AND TITLE-ABS-KEY (({chemotherapy s} OR {drug} OR {therapy} OR {chemotherapies} OR {drug therapy} OR {chemotherapy} OR {cancer treatment} OR {radiation therap*} OR {oncological therap*} OR {radiate} OR {radiated} OR {radiates} OR {radiating} OR {radiation} OR {radiation} OR {electromagnetic radiation} OR {electromagnetic} OR {electromagnetic radiation} OR {radiations} OR {radiation s} OR {radiator} OR {radiators} OR {radiation injur*} OR {therap*} OR {radiotherapy} OR {therapy} OR {antineoplastic agents} OR {radiation} OR {electromagnetic radiation} OR {therapeutics})) AND TITLE-ABS-KEY (({child} OR {children} OR {child s} OR {children s} OR {childrens} OR {childs} OR {pre schooler*} OR {pre schooler*} OR {childhood} OR {childhoods} OR {pre teen*} OR {preteen*} OR {teen*} OR {teenager} OR {adolescent} OR {adolescent} OR {youth} OR {youths} OR {youth s} OR {paediatric patient*} OR {adolescen*} OR {paediatric population} OR {child} OR {child, preschool}] OR {child, preschool} OR {child} OR {preschool}) OR {preschool child} OR {preschooler} OR {preschoolers} OR {preschool} OR {preschooler s} OR {preschools} OR {adolescent}) AND TITLE-ABS-KEY (({cancer s} OR {cancerated} OR {canceration} OR {cancerization} OR {cancerized} OR {cancerous} OR {neoplasms} OR {neoplasms} OR {cancer} OR {cancers} OR {cysts} OR {cysts} OR {cyst} OR {neurofibroma} OR {neurofibroma} OR {neurofibromas} OR {tumor s} OR {tumoral} OR {tumorous} OR {tumour} OR {neoplasms} OR {neoplasms} OR {tumor} OR {tumour s} OR {tumoural} OR {tumourous} OR {tumours} OR {tumors} OR {neoplasms})) AND NOT TITLE-ABS-KEY (({review} OR {systematic review} OR {meta analysis} OR {case report}))) AND PUBYEAR > 2002 AND (LIMIT-TO

	(EXACTKEYWORD , "Human")) AND (LIMIT-TO (LANGUAGE , "English") OR LIMIT-TO (LANGUAGE , "Portuguese"))
Web of Science	("oral mucositis" OR "oral cavity" OR "oral mucosa" OR "oral complications" OR "oral mucosa injuries" OR stomatitis) AND (children OR "pre-schooler*" OR "pre schooler*" OR childhood OR "pre teen*" OR "preteen*" OR teen* OR teenager* OR youth OR "paediatric patient*" OR adolescen* OR "paediatric population" OR child OR "child preschool" OR preschool) AND (Chemotherapy OR "cancer treatment" OR "cancer therap*" OR "drug therap*" OR "radiation therap*" OR "oncological therap*" OR radiation OR "radiation injur*" OR "therap* Radiotherapy" OR "antineoplastic agents" OR Therapeutics) AND (cancer OR tumour OR neoplasms)

Supplementary Table S2. Inclusion and exclusion criteria.

Inclusion	Pediatric patients (≤ 18 years of age)
	Completed or undergoing oncologic treatment
	Presenting outcomes for an oral mucositis therapeutic intervention
Exclusion	Systematic reviews and meta-analysis, narrative reviews, case reports, opinion articles, conference abstracts
	Studies in animal models or *in vitro*
	Reports of children that did not receive cancer treatment
	Reports of children that did not develop oral mucositis
	No defined age range or patients ≥ 19 years of age
	No control group (without intervention or standard oral care)

Supplementary Table S3. Quality assessment analysis using the Modified Newcastle-Ottawa Scale.

**Author and publication year**	**Selection (****)**	**Comparability (**)**	**Exposure (***)**	**Total score Risk: ≤5 = high; 6-7 = moderate; ≥8 = low**
Abdulrhman M *et al.,* 2012	****	**	***	9

Alkhouli M *et al.*, 2021	****	*	***	8
Alkhouli M *et al.*, 2019	****	*	***	8
Amadori F *et al.*, 2016	****	*	**	7
Badr LK *et al.*, 2023	****	**	**	8
Bardellini E *et al.*, 2016	***	*	***	7
Bostanabad MA *et al.*, 2018	***	*	***	7
Chang YH *et al.*, 2017	**	*	**	5
Costa EM *et al.*, 2003	**	*	*	4
Cruz LB *et al.*, 2007	***	**	**	7
de Castro JFL *et al.*, 2013	***	*	*	5
de Koning BA *et al.*, 2007	***	**	***	8
Funato M *et al.*, 2018	*	*	**	4
Gandemer V *et al.*, 2007	****	*	**	7
Gobbo M *et al.*, 2018	****	*	***	8
Gutiérrez-Vargas R *et al.*, 2020	***	*	***	7
Immonen E *et al.*, 2020	***	*	**	6
Kamsvåg T *et al.*, 2020	*	*	**	4
Khurana H *et al.*, 2013	***	**	**	7
Kobya HB *et al.*, 2016	***	**	**	7
Lauritano D *et al.*, 2014	*	**	**	5
Lucchese A *et al.*, 2016	****	*	***	8
Medeiros-Filho JB *et al.*, 2017	***	**	***	8
Morris J *et al.*, 2016	**	*	**	5
Mubaraki S *et al.*, 2020	****	**	**	8
Nunes LFM *et al.*, 2020	**	**	***	7
Pinto LP *et al.*, 2006	**	*	*	4
Prakash S *et al.*, 2020	****	*	***	8

Raphael MF *et al.,* 2014	***	*	***	7
Rathe M *et al.,* 2020	****	*	***	8
Reyad F *et al.,* 2022	****	*	***	8
Sato A *et al.*, 2006	**	**	***	7
Shah D *et al.,* 2023	****	*	***	8
Shahrabi M *et al.,* 2022	***	*	***	7
Soares ADS *et al.,* 2021	***	*	***	7
Soto M *et al.,* 2015	**	*	*	4
Sung L *et al.,* 2007	***	*	**	6
Vitale M *et al.,* 2017	****	*	**	7
Widjaja NA *et al.,* 2020	***	**	***	8

Supplementary Table S4. Additional characteristics of the included studies.

Author and publication year	Oral mucositis grade (WHO scale)	Oral mucositis treatment dose	Most common malignant disease	Antineoplastic intervention details
Abdulrhman M *et al.,* 2012	2 - 3	0.5 g/kg (maximum 15 g) 3 times daily<br><br>or<br><br>0.25 g/kg (maximum 5 g) 3 times daily	ALL	Methotrexate (2 g/m^2)
Alkhouli M *et al.*, 2021	0	70% solution 2 times daily	ALL	(n.a.)
Alkhouli M *et al.,* 2019	0	5 ml	ALL	Methotrexate
Amadori F *et al.,* 2016	2 - 4	830 nm, 150 mW, 1 cm^2, 30 s per cm^2, 4.5 J/cm^2	(n.a.)	(n.a.)
Badr LK *et al.,* 2023	1 - 4	2.5 cc	ALL	Doxorubicin or Methotrexate
Bardellini E *et al.,* 2016	1 - 2	15 mL 3 times daily	ALL	(n.a.)
Bostanabad MA *et al.,* 2018	1 - 2	(n.a.)	ALL and AML	Cisplatin, Cyclophosphamide, Methotrexate, Adriamycin, or Vincristine
Chang YH *et al.,* 2017	1 - 2	0.4 g/kg/day	ALL	Methotrexate (2.5 g/m^2 or 5 g/m^2)
Costa EM *et al.,* 2003	(n.a.)	(n.a.)	ALL	6-mercaptopurine (50 mg/m^2/day) AND

				Methotrexate (2 mg/m^2)
de Castro JFL *et al.,* 2013	(n.a.)	660 or 830 nm, 100 mW, 3.57 W/cm^2, 0.028 cm^2, 1 J	ALL	Methotrexate (2.5 g/m^2 or 5 g/m^2)
de Koning BA *et al.,* 2007	3 - 4	62 µg TGF- β2/day (<20 kg) or 124 µg TGF- β2/day (20-40 kg) or 186 µg TGF-β2/day (>40kg)	ALL, AML, NHL	Vincristine, Cyclofosfamide, Doxorubicine,High dose-Methotrexate
Funato M *et al.,* 2018	0	5 ml 4 times daily	Neuroblastoma	Carboplatin, Melphalan, and Etoposide
Gandemer V *et al.,* 2007	3 - 4	5 or 6 pieces per day	Lymphoma	Cyclophosphamide-Vincristine-Prednisone-Methotrexate (3 g/m^2 and 8 g/m^2)-Doxorubicin
Gobbo M *et al.,* 2018	3 - 4	660 and 970 nm, 3.2 W, 320mW/cm^2,36.8J/cm^2	ALL	(n.a.)
Gutiérrez-Vargas R *et al.,* 2020	0	50 mg/day	ALL and AML	(n.a.)
Immonen E *et al.,* 2020	0	(n.a.)	ALL	Methotrexate (≥1 g/m^2)
Kamsvåg T *et al.,* 2020	0	≥ 30 min in 74 sessions	ALL	Busulfan
Khurana H *et al.,* 2013	0 - 4	200 mg/day	ALL, AML, NHL	Mitoxantrone, Chlorambucil, and Prednisolone
Kobya HB *et al.,* 2016	1 - 4	1 g/kg/day	ALL	St Jude Total XV protocol
Lauritano D *et al.,* 2014	1 - 4	60 mg/kg/day	ALL	12 Gy over eight sittings of 150 cGy twice a day for 4 days
Lucchese A *et al.,* 2016	(n.a.)	60 µg/kg	ALL	(n.a.)
Medeiros-Filho JB *et al.,* 2017	(n.a.)	Photosensitizer for 5 min and 660 nm, 100 mW, 1 cm above the lesion, and 90 s	(n.a.)	(n.a.)
Morris J *et al.,* 2016	(n.a.)	60 µg/kg/day or 80 µg/kg/day	ALL	1200 cGy, Etoposide (1500 mg/m^2), Cyclo-phosphamide (120 mg/kg)
Mubaraki S *et al.,* 2020	(n.a.)	4 times daily	ALL and AML	Fludarabine, Busulphan, Cyclophosphamide, Methotrexate, Cyclosporine, or Antithymocyte globulin
Nunes LFM *et al.,* 2020	0	660 nm, 100 mW, 2 J, 3.33 W/cm2, and 20 s	ALL	Methotrexate

Pinto LP *et al.,* 2006	0	Cotton pad immersed in the solution for 1 min	ALL	Methotrexate
Prakash S *et al.,* 2020	3 - 4	1 mg/kg (maximum 40 mg)	ALL	(n.a.)
Raphael MF *et al.,* 2014	0 - 4	4 times daily	ALL	(n.a.)
Rathe M *et al.,* 2020	0	0.5-1 g/kg/day	ALL	Nordic Society of Paediatric Haematology and Oncology ALL 2008 protocol
Reyad F *et al.,* 2022	1 - 4	980 nm, 1.5 W, 30 s, 4.5 J/cm^2	ALL	(n.a.)
Sato A *et al.*, 2006	0	1.5 mg/kg/day	Solid tumors	Etoposide (200 mg/m^2), Carboplatin (400 mg/m^2), Melphalan (60 mg/m^2) and Pirarubicin hydro-chloride (40 mg/m^2)
Shah D *et al.,* 2023	0	1 mg/kg/day	ALL	(n.a.)
Shahrabi M *et al.,* 2022	0	Spray every 6 hours	ALL	Cyclophosphamide (60 mg/kg/day)
Soares ADS *et al.,* 2021	(n.a.)	Application every 6 hours	ALL	(n.a.)
Soto M *et al.,* 2015	0 - 4	830 nm, 80 mW, 30 sec, 2.4 J	ALL and AML	Busulfan and Melphalan
Sung L *et al.,* 2007	0	2 mL/day	Ewing's sarcoma	Doxorubicin (60 mg/m^2)
Vitale M *et al.,* 2017	4	970 nm, 3.2 W, 230 s	(n.a.)	(n.a.)
Widjaja NA *et al.,* 2020		400 mg/kg/day	ALL	2013 Indonesian ALL Chemotherapy Protocol

n.a., not available; ALL, Acute Lymphoblastic Leukaemia; AML, Acute Myeloid Leukemia; NHL, non-Hodgkin Lymphoma.
